# Investigation of sheath properties in a warm plasma with two kappa-distributed electrons and monoenergetic electron beam

**DOI:** 10.1038/s41598-022-08436-1

**Published:** 2022-03-17

**Authors:** M. M. Hatami

**Affiliations:** Physics Department of K. N. Toosi University of Technology, Tehran, 15418-49611 Iran

**Keywords:** Plasma physics, Space physics

## Abstract

Sheath formation criterion of an electropositive plasma consisting of singly charged positive ions, two kappa-distributed electron species with different effective temperatures and a monoenergetic electron beam is investigated by the Sagdeev potential approach. Using this criterion, effects of electron beam, superthermality of electron species as well as temperature and concentration of positive ions on the sheath properties are studied numerically. It is shown that the temperature of positive ions, concentration and superthermality of electron species and presence of electron beam affect Bohm velocity of positive ions. Also, it is observed that density distribution of the charged particles and sheath thickness increase in the presence of electron beam. In addition, it is found that with increasing the ion temperature, the sheath width and density distribution of the charged particles in the sheath area decrease.

## Introduction

Many observations have confirmed the existence of both cool and hot electron populations with a non-Maxwell distribution function^1–4^. For example, analysing data from Cassini spacecraft by Schippers et al.^4^ showed a best fit for both cool and hot electron velocity distributions with kappa distributions with low values of $\kappa$. Although the empirical evidence for the kappa distributions function dates back to the 1960s, its statistical justification occurred in the 1980s based on maximizing the q-entropy^5–7^. Kappa distributions may be produced in space and astrophysical plasmas by various mechanisms such as superstatistics^8,9^, the effect of shock waves^10^, pump acceleration mechanism^11^ and etc.

The power law one dimensional kappa velocity distribution is given by:

1 $$\begin{aligned} f_{\kappa }(v)=\left( \frac{n_0\Gamma (\kappa )}{\Gamma (\kappa -1/2) (\pi \kappa \theta ^2)^{1/2}}\right) \left( 1+\frac{v^2}{\kappa \theta ^2}\right) ^{-\kappa }, \end{aligned}$$

where $f_{\kappa }(v)$ is distribution function, $n_0$ is the equilibrium density, $\Gamma$ is the standard gamma function, $\kappa$ is the parameter of the superthermal particles and is known as spectral index, $\theta =\bigg (\displaystyle \frac{2\kappa -3}{\kappa }\displaystyle \frac{k_BT}{m_p}\bigg )^{1/2}$ denotes the effective thermal speed or most probable speed of the particles of mass $m_p$ and temperature *T* and, $k_B$ is the Boltzmann constant. From $\theta$, we note that a well-defined value of effective thermal speed requires $\kappa >3/2$^12^. Integrating the kappa distribution over velocity space, the electron number density can be written as follows:

2 $$\begin{aligned} n_{e}(\varphi )=n_{0e}\left( 1-\frac{e\varphi }{(\kappa _e-3/2)k_BT_e} \right) ^{-\kappa _e+1/2}, \end{aligned}$$

where $n_{0e}$ and $T_e$ are the equilibrium number density and temperature of electrons, respectively, $\kappa _e>3/2$ is the spectral index and $\varphi$ is the electrostatic potential.

During the last decade, kappa distribution has been used by many authors for various research purposes such as occurrence and propagation of electron-acoustic and ion-acoustic waves, dust-ion-acoustic shock waves and electromagnetic cyclotron instabilities in plasma with two kappa-distributed electron species^13–22^. On the other hand, sheath formation in plasmas is one of the fundamental issues in plasma physics. Recently, there has been a lot of interest in the study of plasma sheath formation in the presence of non-Maxwellian electrons^23–31^. As a result of these studies, it has been found that depending on the degree of superthermality of electrons, the velocity of the ions at the sheath edge could be more or less than their velocity in the corresponding plasma with Maxwellian electrons. Therefore, it can be concluded that the presence of non-Maxwellian electrons makes a significant contribution to the variation of plasma sheath properties.

Since electrons have a significant effect on the plasma sheath layer, the study of this layer in the presence of an electron beam, anisotropic distributed electrons, etc. is very important. For this reason, the effect of presence of an electron beam on plasma sheath properties has been the subject of many studies^33–39^. In 1990, Ingram and Braithwaite have used a quasi-neutral model to examine the nature of the plasma-sheath boundary in the presence of a high-energy monoenergetic electrons and Maxwellian electrons^32^. The behavior of the plasma sheath boundary in the presence of an electron beam with finite temperature has been examined by Bradley and Amemiya^33^. Pal et al.^34^ have experimentally investigated the effect of an electron beam on plasma characteristics and boundary sheath in a dc discharge plasma. Demidov et al.^35^ have discussed the sheath formation in plasmas with low density nonlocal fast electrons. Sharifian and Shokri^36^ have studied the influence of the presence of a fast monoenergetic electron beam on the temporal evolution of the ion-matrix sheath in an electropositive plasma with Maxwellian electrons. Gyergyek et al.^37^ have presented a fluid model of the sheath formation in front of a planar electron emitting electrode immersed in a plasma with Maxwellian electrons and a monoenergetic electron beam. Chekour et al.^38^ have investigated the Bohm criterion in an electronegative dusty plasmas in the presence of a fast monoenergetic electrons and Maxwellian distributed electrons and negative ions. Ou et al.^39^ have presented a sheath model to investigate the plasma-wall interaction in the fusion boundary layer in the presence of a fast monoenergetic electron beam.

In 2012, Badman et al.^40^ reported the existence of electron beams at Saturn based on Cassini observations. Also, as mentioned earlier, the analysis of Cassini data revealed the existence of two species of electrons (cool and hot) with a kappa distribution function in Saturn^4^. These facts encourage us to study the structure of plasma sheath in a plasma consisting of fluid positive ions and two kappa-distributed electrons with different effective temperatures in the presence of a monoenergetic electron beam which, to our knowledge, has not yet been investigated. Therefore, the main focus of the current work is to discuss about the effect of presence of an electron beam on the sheath properties of a multi-component electropositive plasma containing ions and two kappa-distributed electron species with different effective temperatures. In order to do this, we first derive the sheath formation criterion for the mentioned plasma system and then we investigate the behavior of the plasma sheath characteristics in the presence of an electron beam. The results of the present work should be useful in understanding the properties of plasma sheath in space plasmas, such as Saturn’s magnetosphere^13^ and Pulsar magnetosphere^41^ where the combined presence of electron beams and excess superthermal electrons may be encountered.

This work is arranged in four sections including the introduction as the first section. In “Model and basic equations” section, the basic equations of the considered plasma are presented. Bohm sheath criterion and the plasma sheath characteristics are investigated in “Results and discussion” section, and finally, our brief conclusion is presented in “Conclusion” section.

## Model and basic equations

**Figure 1:**
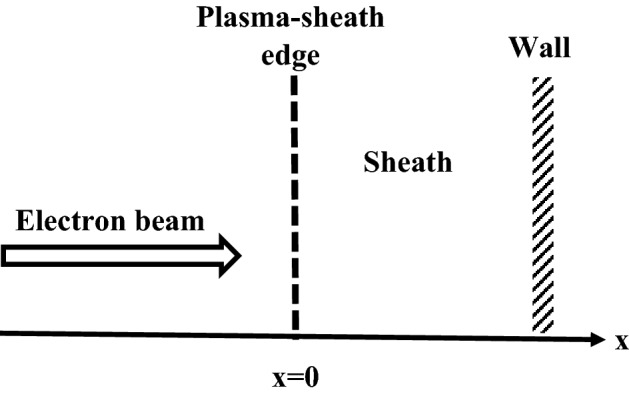
Schematic geometry of the sheath model.

Let us consider an electropositive plasma containing warm fluid ions and two kappa-distributed electron species with two different effective temperatures in the presence of an electron beam. Assuming the *x* axis to be normal to the wall and the physical parameters change only along the *x* (see Fig. 1), the fluid equations for the warm, collisionless ions are described as

3 $$\begin{aligned} \frac{d}{dx}( n_{i}v_{i})= & {} 0, \end{aligned}$$

4 $$\begin{aligned} m_iv_{i}\frac{d v_{i}}{d x}= & {} -e\frac{d\varphi }{dx}-\frac{1}{n_i}\frac{d p_i}{dx}, \end{aligned}$$

where *e* is the unit electric charge, $\varphi$ is the electrostatic potential and $n_i$, $v_i$, $m_i$ and $p_i$ are density, velocity, mass and pressure of positive ions, respectively. The positive ion pressure $p_i$ is related to the ion density as follows:

5 $$\begin{aligned} p_i={k_BT_{i}n_{i}}^{\gamma _i}/n_{0i}^{\gamma _{i-1}}, \end{aligned}$$

where $T_i$ is the ion temperature, $n_{0i}$ is the ion density at the sheath edge $(x=0)$ and $\gamma _i=3,~2,~5/3$ for unidimensional, bidimensional, or tridimensional adiabatic flow, respectively.

The number densities of kappa-distributed electron species are expressed as:

6 $$\begin{aligned} n_j=n_{0j}\left( 1-\frac{e\varphi }{k_BT_{ej}(\kappa _{j}-3/2)}\right) ^{-\kappa _j+1/2}, \end{aligned}$$

where $j=c,~h$ refer to cool and hot electron species and $T_{ej}$ and $n_{0j}$ are the temperature and density of electron species j at the sheath edge, and $\kappa _j>3/2$ is the spectral index of each electron species.

Similar to Ref.^37^, we assume that there is an isotropic, monoenergetic electron beam with a unidirectional velocity distribution in the plasma medium. Therefore, in addition to the fact that the velocities of all electrons are the same (called $v_b$), the directions of their velocities are uniformly distributed in space. As a result, the velocity distribution of the monoenergetic electrons is defined as follows:

7 $$\begin{aligned} f_b(v)=\displaystyle \frac{n_{0b}}{4\pi v_b^2}\delta (v-v_b), \end{aligned}$$

where $n_{0b}$ is the density of the beam electrons far from the plane wall. Integrating $f_b(v)$ over the velocity space, the density of the beam electrons is written as follows^37^:

8 $$\begin{aligned} n_b(x)=\int _v f_b(v)d^3 v=\frac{n_{0b}}{2}\left( 1-\sqrt{-\frac{2e\varphi (x)}{m_ev^2_b}}\right) . \end{aligned}$$

From relation (8), it is seen that $n_b(x)$ decreases by increasing the electrostatic potential of the sheath layer. Moreover, to avoid complicating the presented fluid model, the effects of the collision of beam electrons in the presheath layer have been neglected.

Finally, the potential in the sheath $\varphi$ is determined by the Poisson equation:

9 $$\begin{aligned} \frac{d^2\varphi }{d x^2}=\frac{e}{\varepsilon _{0}}(n_{c}+n_{h}+n_b-n_{i}), \end{aligned}$$

where $\varepsilon _{0}$ is the electric permittivity of free space. The quasineutrality condition at the sheath edge of such a multi-component plasma is $n_{0i}=n_{0c}+n_{0h}+n_{0b}$.

We normalize the physical quantities in (3)–(6), (8) and (9) with the following normalized variables:

$$\begin{aligned} N_i= & {} n_i/n_{0c},~~N_{j}=n_j/n_{0c},~~ N_b=n_b/n_{0c},~~\sigma _j=T_{ej}/T_{ec},~~ \xi =x/\lambda _{D},~~u_i=v_i/c_{s}, \\ u_e= & {} v_b/c_{s},~~\delta =n_{0h}/n_{0c},~~ \delta _b=n_{0b}/n_{0c},~~ \sigma _i=T_i/T_{ec},~~\phi =-e\varphi /k_BT_{ec},~~ \delta _i=n_{0i}/n_{0c} \end{aligned}$$

where $$j=c, h$$, $$\lambda _{D}=(\varepsilon _{0}k_BT_{ec}/e^2n_{0i})^{1/2}$$ and $$c_s=(k_BT_{ec}/m_i)^{1/2}$$.

Using the above mentioned normalized variables for a unidimensional adiabatic ion flow, the basic equations of our fluid model can be written as follows:

10 $$\begin{aligned}&\frac{d( N_{i}u_{i})}{d\xi }=0, \end{aligned}$$

11 $$\begin{aligned}&u_{i}\frac{du_{i}}{d\xi } =\frac{d\phi }{d\xi }-\frac{3}{2}\left( \frac{\sigma _i}{\delta _i^2}\right) \frac{d N_i^2}{d\xi }, \end{aligned}$$

12 $$\begin{aligned}&\frac{d^2\phi }{d \xi ^2}=\frac{1}{\delta _i}\bigg [N_i-\left( 1+\frac{\phi }{\sigma _c(\kappa _c-\frac{3}{2})}\right) ^{-\kappa _c+1/2}-\delta \left( 1+\frac{\phi }{\sigma _h(\kappa _h-\frac{3}{2})}\right) ^{-\kappa _h+1/2}-\frac{\delta _b}{2}\left( 1-\sqrt{\frac{2\phi }{m u_e^2}}\right) \bigg ], \end{aligned}$$

where $$m=m_e/m_i$$.

## Results and discussion

In this section, we are going to analytically derive the sheath formation criterion for an electropositive plasma consisting of singly charged positive ions and two kappa-distributed electron species in the presence of a monoenergetic electron beam. Without knowing this criterion, it will not be possible to use the normalized basic equations to numerically study the sheath structure in the considered plasma.

Multiplying the normalized Poisson equation by $(d\phi /d\xi )$ and integrating, we obtain

13 $$\begin{aligned} \left( \frac{d \phi }{d \xi }\right) ^2=\left( \frac{d \phi }{d \xi }\right) _{\xi =0}^2-2S(\phi ,u_{0i}), \end{aligned}$$

where

14 $$\begin{aligned} S(\phi ,u_{0i})= & {} -u_{0i}\int ^\phi _{\phi _0}\displaystyle \frac{d\phi }{u_i}-\frac{\sigma _c}{\delta _i}\left( 1+\displaystyle \frac{\phi }{\sigma _c\left( \kappa _c-\displaystyle \frac{3}{2}\right) }\right) ^{-\kappa _c+3/2}\bigg |_{\phi _0}^\phi \nonumber \\&-\frac{\sigma _h\delta }{\delta _i}\left( 1+\displaystyle \frac{\phi }{\sigma _h\left( \kappa _h-\displaystyle \frac{3}{2}\right) }\right) ^{-\kappa _h+3/2}\bigg |_{\phi _0}^\phi +\left( \frac{\delta _b}{2\delta _i}\phi -\displaystyle \frac{\delta _b}{3\delta _i}\sqrt{\displaystyle \frac{2}{m u_e^2}}\phi ^{3/2}\right) \bigg |_{\phi _0}^\phi , \end{aligned}$$

is the Sagdeev potential and $\phi _0$ and $u_{0i}$ are the normalized electrostatic potential and normalized velocity of the positive ions at the sheath edge $(\xi =0)$, respectively.

From (14), it is found that $S(\phi _0,u_{0i})=0$ and $\partial S(\phi _0,u_{0i})/\partial \phi =0$. Therefore, the sheath edge is an extremum point for the Sagdeev potential. However, it should be mentioned here that the concept of sheath edge resulting from the Bohm criterion is suitable for collisionless plasmas^42^. Maximizing *S* in the sheath edge, we obtain

15 $$\begin{aligned} \displaystyle \frac{\partial ^2 S(\phi _0,u_{0i})}{\partial \phi ^2}= \displaystyle \frac{1}{u_{0i}^2-3\sigma _i}-\displaystyle \frac{\delta _b}{4\delta _i}\sqrt{\displaystyle \frac{2}{m u_e^2\phi _0}}-\displaystyle \frac{1}{\delta _i}(A+B)\le 0, \end{aligned}$$

or

16 $$\begin{aligned} u_{0i}\ge \left( 3\sigma _i+\displaystyle \frac{\delta _i}{A+B +\displaystyle \frac{\delta _b}{4}\sqrt{\displaystyle \frac{2}{m u_e^2\phi _0}}}\right) ^{1/2}, \end{aligned}$$

where

17 $$\begin{aligned} A=-\displaystyle \frac{1}{\sigma _c}\left( \frac{\kappa _c-\displaystyle \frac{1}{2}}{\kappa _c-\displaystyle \frac{3}{2}}\right) \left( 1+\displaystyle \frac{\phi _0}{\sigma _c\left( \kappa _c-\displaystyle \frac{3}{2}\right) }\right) ^{-\kappa _c+1/2}, \end{aligned}$$

and

18 $$\begin{aligned} B=-\frac{\delta }{\sigma _h}\left( \frac{\kappa _h-\displaystyle \frac{1}{2}}{\kappa _h-\displaystyle \frac{3}{2}}\right) \left( 1+\displaystyle \frac{\phi _0}{\sigma _h\left( \kappa _h-\displaystyle \frac{3}{2}\right) }\right) ^{-\kappa _h+1/2}. \end{aligned}$$

Using the normalized quasineutrality condition at the sheath edge, $\left( N_c+N_h+N_b=N_i\right) _{\phi =\phi _0}$, it is easily found that

19 $$\begin{aligned} \delta _i=\left( 1+\frac{\phi _0}{\sigma _c(\kappa _c-\frac{3}{2})}\right) ^{-\kappa _c+1/2}+\delta \left( 1+\frac{\phi _0}{\sigma _h(\kappa _h-\frac{3}{2})}\right) ^{-\kappa _h+1/2}+\frac{\delta _b}{2}\left( 1-\sqrt{\frac{2\phi _0 }{m u_e^2}}\right) . \end{aligned}$$

Relation (16) is the sheath formation criterion for an electropositive plasma consisting of warm positive ions, two species of kappa-distributed electrons and a monoenergetic electron beam. From (16), it is found that the Bohm velocity of positive ions at the sheath edge depends on different plasma parameters such as temperature and initial (unperturbed) density of ions and electron species, spectral index of each electron species, concentration and velocity of the electron beam and electrostatic potential at the sheath edge. Examining Eq. (16) for some specific cases can be useful. For example, ignoring the presence of electron beam and assuming $\phi _0=0$, relation (16) leads to the ion velocities at the sheath edge of an electropositive plasma with two species of kappa-distributed electrons^14^. Also, assuming $\delta _b=0$ in the limit $\kappa _{c,h}\rightarrow \infty$, the modified sheath criterion (Eq. (16)) reduces to $u_{0i}\ge \left( 1+3\sigma _i\right) ^{1/2}$ for $\phi _0=0$ which is the sheath criterion for an ion-electron plasma with warm ions and Maxwellian-distributed electrons^43^. Finally, assuming that the ions are cold and the electrons are in thermal equilibrium ($\kappa _{c,h}\rightarrow \infty$), Eq. (16) will lead to the well-known Bohm criterion $u_{0i}\ge 1$ in the absence of the electron beam^44^.

Using Eqs. (10)–(12) and (16), one can study the plasma sheath properties of an electropositive plasma consisting of warm fluid positive ions and two species of kappa-distributed electrons with different effective temperature in the presence of a monoenergetic electron beam. Before doing so, we are going to investigate the behavior of the modified Bohm criterion with different plasma parameters.

**Figure 2:**
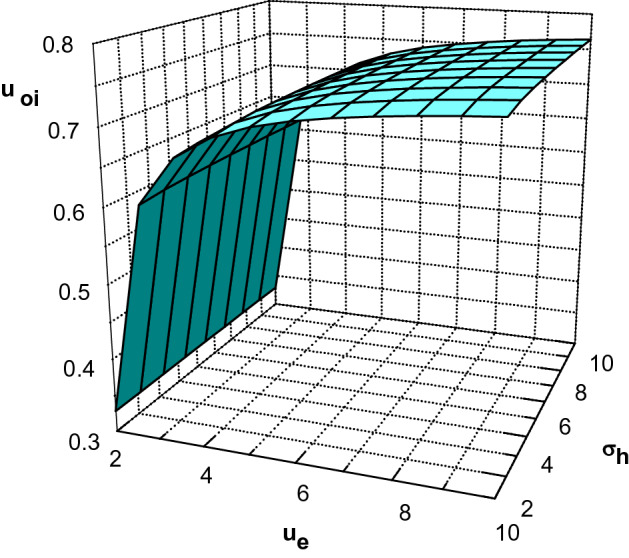
Ion velocities at the sheath edge as a function of $u_e$ and $\sigma _h$ for $\phi _0=0.008$, $\kappa _c=2.4$, $\kappa _h=4$, $\sigma _i=0.02$, $m=1/1836$, $\delta =0.2$ and $\delta _b=0.001$.

Figure 2 shows variation of minimum velocity of the positive ions at the sheath edge $u_{0i}$ with the electron beam velocity $u_e$ and effective temperature ratio of hot-to-cool electron species $\sigma _h$ for $\phi _0=0.008$, $\kappa _c=2.4$, $\kappa _h=4$, $\sigma _i=0.02$, $\delta =0.2$ and $\delta _b=0.001$. Hereafter, to prevent the divergence of the second term in (16), we assume that $\phi _0$ takes a nonzero but infinitesimal value in our numerical calculation. It is seen that an increase in beam velocity has a negligible effect on the ion velocities at the sheath edge. Also, similar to plasma with two nonextensive electron species^27^, it is observed that $u_{0i}$ increases by increasing the effective temperature ratio of hot-to-cool electron species.

**Figure 3:**
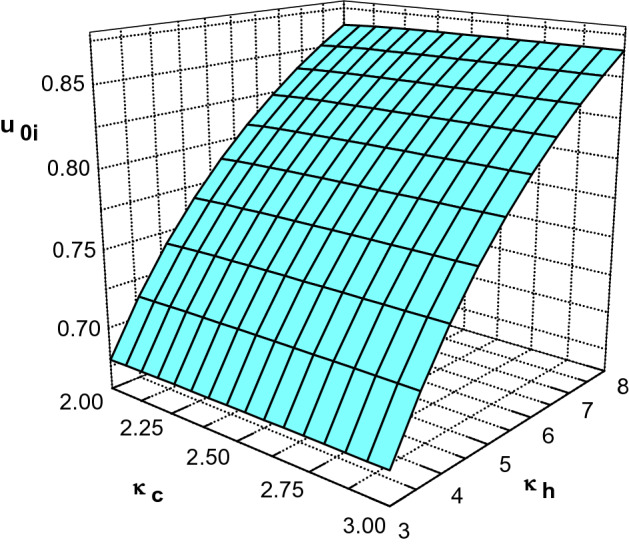
Ion velocities at the sheath edge as a function of $\kappa _c$ and $\kappa _h$ for $\phi _0=0.008$, $\sigma _h=10$, $\sigma _i=0.02$, $m=1/1836$, $\delta =0.2$ and $\delta _b=0.001$.

Variation of $u_{0i}$ with the spectral index of cool $\kappa _c$ and hot $\kappa _h$ electron species is shown in Fig. 3 for $\phi _0=0.008$, $\sigma _h=10$, $\sigma _i=0.02$, $\delta =0.2$ and $\delta _b=0.001$. Considering the typical range of $\kappa _c$ and $\kappa _h$^4^, it is seen that $u_{0i}$ increases by increasing the spectral index of each electron species. As we know, increasing $\kappa _{c,h}$ causes the superthermal particles to decrease and therefore an increase in superthermality leads to lower $u_{0i}$. Similar result has been reported for plasmas without electron beam^14^.

**Figure 4:**
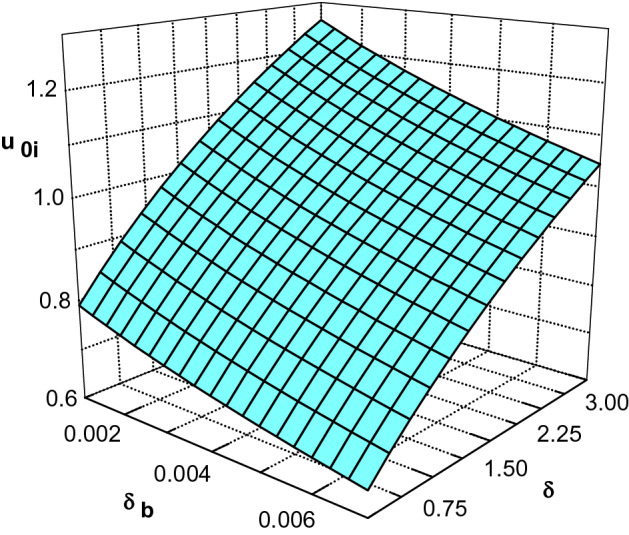
Ion velocities at the sheath edge as a function of $\delta$ and $\delta _b$ for $\phi _0=0.008$, $\sigma _h=10$, $\sigma _i=0.02$, $m=1/1836$, $\kappa _c=2.4$ and $\kappa _h=4$.

Effect of hot-to-cool electron initial densities $\delta$ and the beam density $\delta _b$ on the modified sheath formation criterion is indicated in Fig. 4 for $\kappa _c=2.4$, $\kappa _h=4$ and the other parameters of Fig. 3. One can see that any increase in density of hot electron species leads to an increase in the Bohm velocity of positive ions but the beam density has a different effect on the behavior of $u_{0i}$ and as can be seen, with increasing $\delta _b$, the Bohm velocity decreases.

**Figure 5:**
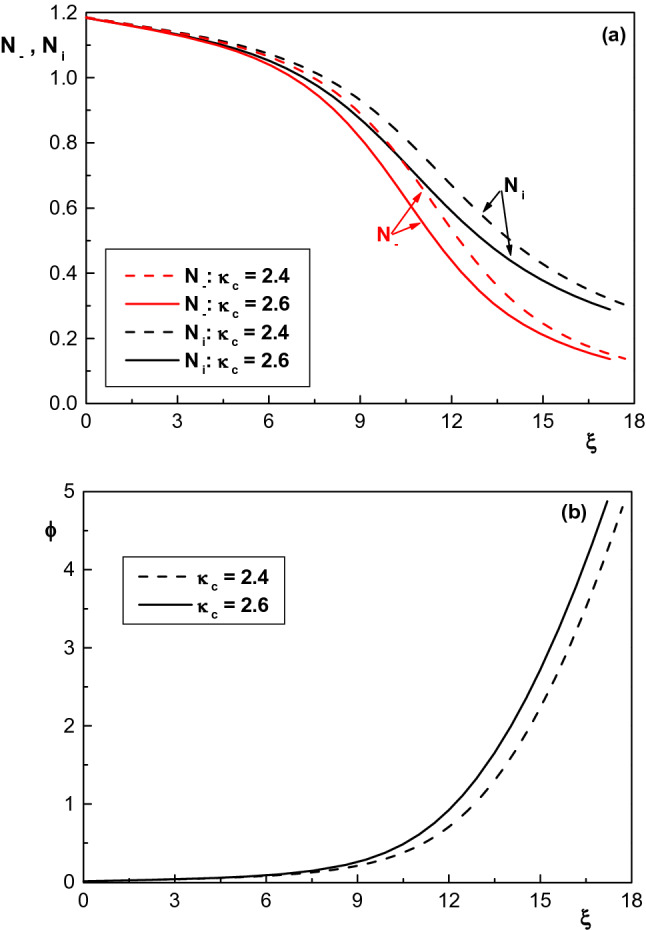
Spatial distribution of (**a**) normalized density of positive and negative particles and (**b**) normalized sheath potential for $\phi _0=0.008$, $\sigma _h=10$, $\sigma _i=0.02$, $m=1/1836$, $\kappa _h=4$, $\delta =0.2$, $\delta _b=0.002$ and different values of $\kappa _c$.

Variation of density distribution of the charged particles ($N_i$ and $N_-=N_c+N_h+N_b$) and electrostatic potential of the sheath region with the spectral index of cool electron species $(\kappa _c)$ is illustrated in Fig. 5a,b, respectively for $\phi _0=0.008$, $\sigma _h=10$, $\sigma _i=0.02$, $\delta =0.2$, $\kappa _h=4$ and $\delta _b=0.002$. Here, due to the similarity of the results, we have ignored the study of variation in $N_i$, $N_-$ and $\phi$ with $\kappa _h$. Figure 5a shows density distribution of negative particles $N_-$ decrease with $\kappa _c$. Given the fact that the number of fast electrons decreases with increasing the spectral index of electron species (e.g., $\kappa _c$), the decrease in negatively charged particle densities is understandable. Normalized density distribution of positive ions also decreases with increasing $\kappa _c$. As explained, any increase in $\kappa _c$ causes a decrease in the population of energetic electrons. The result is a reduction in the number of electrons reaching the wall which in turn reduces the floating potential, and thus the number of ions. Moreover, Fig. 5b shows that the potential of the sheath also increases as $\kappa _c$ increases which is due to decrease of floating potential with $\kappa _c$.

**Figure 6:**
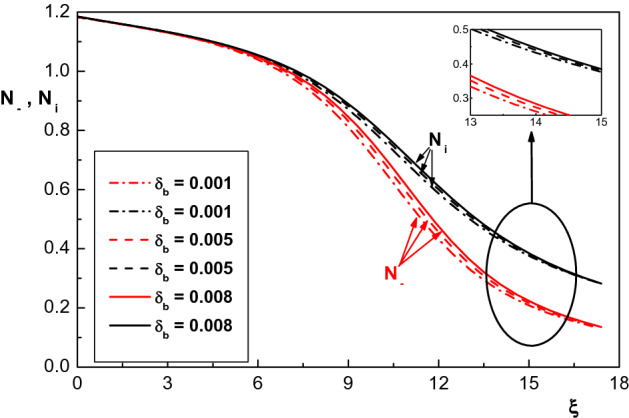
Spatial distribution of the normalized density of positive ions and negatively charged particles for $\kappa _c=2.4$ and different values of $\delta _b$. The other parameters are the same with Fig. 5.

Now, we are going to investigate the effect of presence of monoenergetic electron beam ($\delta _b$) on the properties of the sheath region of an ion-electron plasma with two species of kappa-distributed electrons. Figure 6 indicates variation of density distribution of positive ions with the beam density for the same parameters of Fig. 5. This figure shows that an increase in $\delta _b$ leads to an increase in density distribution of positive ions. Also, this figure shows variation of density distribution of negatively charged particles $N_-$ with different values of $\delta _b$ which also increases with increasing $\delta _b$. This can be explained by the fact that increasing the beam density increases the number of electrons reaching the cathode and thus increases the cathode potential. Therefore, the density of ions also increases with increasing $\delta _b$. These results are consistent with those reported in Refs.^35,36^ for a plasma with Maxwellian electrons and monoenergetic electron beam.

**Figure 7:**
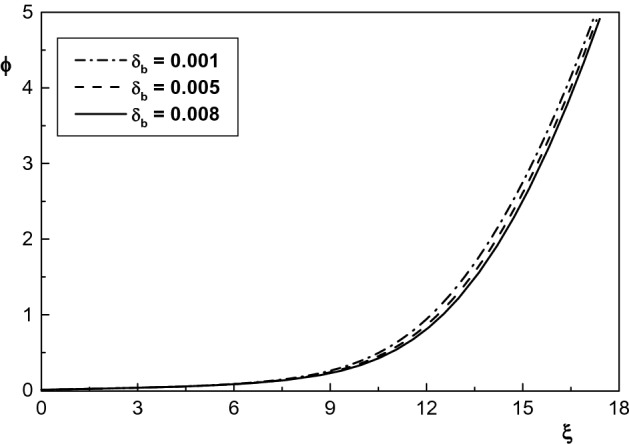
Spatial distribution of the normalized sheath potential for $\kappa _c=2.4$ and different values of $\delta _b$. The other parameters are the same with Fig. 5.

Effect of beam density on the electrostatic potential of the sheath region is depicted in Fig. 7. From this figure one can see that an increase in the beam density causes a decrease in the potential of sheath. Also, it is seen that the sheath thickness increases by increasing $\delta _b$ which is in agreement with the result of Refs.^35,36^. The reason behind this is that the potential of the wall increases by increasing $\delta _b$ and as a result the sheath width decreases. Therefore, it can be concluded from Figs. 6 and 7 that density distribution of negatively charged particles and positive ions in the sheath increases in the presence of electron beam while the potential of the sheath region decreases.

**Figure 8:**
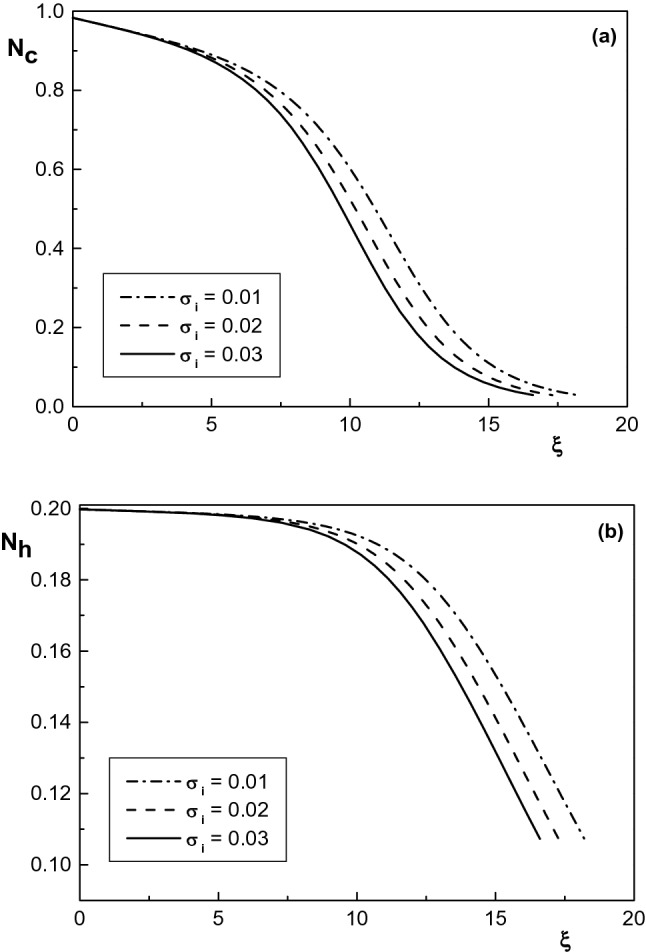
Spatial distribution of the normalized density of (**a**) cool and (**b**) hot electron species for $\kappa _c=2.4$, $\delta _b=0.005$ and different values of $\sigma _i$. The other parameters are the same with Fig. 5.

**Figure 9:**
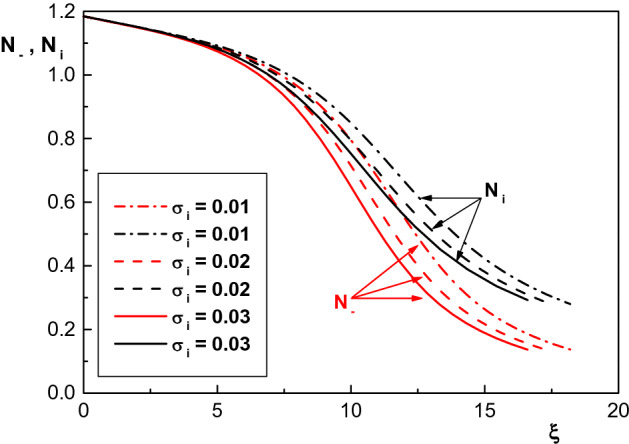
Spatial distribution of the normalized density of positive ions and negatively charged particles for $\kappa _c=2.4$, $\delta _b=0.005$ and different values of $\sigma _i$. The other parameters are the same with Fig. 5.

At the end of this section, we study the effect of temperature of positive ions on the sheath properties of an electropositive plasma containing monoenergetic electron beam, warm positive ions and two kappa-distributed electron species. Figure 8a,b show the effect of positive ion temperatures on the density distribution of cool and hot electron species in the sheath region for $\delta =0.2$, $\delta _b=0.005$, $\kappa _c=2.4$, $\kappa _h=4$, $\phi _0=0.008$, $\sigma _h=10$ and different values of $\sigma _i$. It is seen that density of both electron species decreases as the ion temperature increases. In addition, Fig. 9 shows that the behavior of density distribution of the negatively charged particles for different values of $\sigma _i$ is similar to Fig. 8 and $N_-$ decreases by increasing the positive ion temperatures. It is also seen that the positive ion density $N_i$ is falling, too, which is due to the ion acceleration. A similar result has previously been reported in Refs.^43,45^ for a warm Maxwellian plasma.

**Figure 10:**
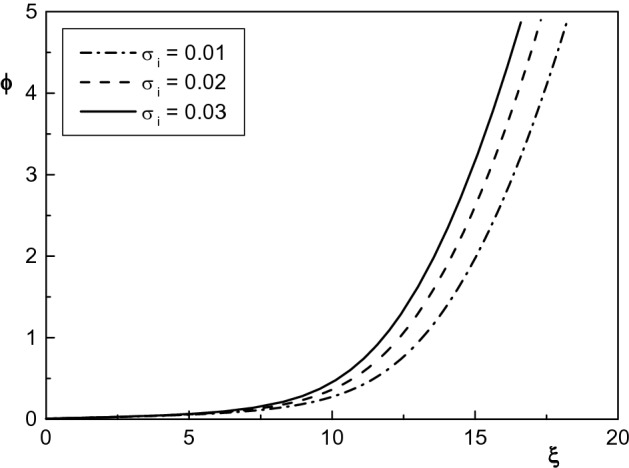
Spatial distribution of the normalized sheath potential for $\kappa _c=2.4$, $\delta _b=0.005$ and different values of $\sigma _i$. The other parameters are the same with Fig. 5.

Finally, the effect of temperature of positive ions on the normalized potential of the sheath region of a plasma with two kappa-distributed electrons and electron beam is depicted in Fig. 10. It is seen that the normalized potential increases by increasing $\sigma _i$ in the presence of electron beam. Also, in confirming the results of previous works, it is seen that the sheath width decreases with increasing ion temperature^46^. The reason for this is that as the temperature of the ions increases, $u_{0i}$ and consequently the velocity of the ions hits the cathode increases. As a result, the sheath thickness decreases by increasing $\sigma _i$. Therefore, it can be concluded that density distribution of the charged particles decreases by increasing $\sigma _i$ while electrostatic potential of the plasma sheath region increases.

## Conclusion

Sheath formation criterion was investigated in a collisionless electropositive plasma with a monoenergetic electron beam by using the hydrodynamic equations. It was assumed that the plasma consisting of singly charged positive ions with finite temperature and two species of kappa-distributed electrons with different effective temperatures. Using Sagdeev potential approach, the modified Bohm criterion was derived and it was shown that the ion velocity at the sheath edge depends on various plasma parameters such as spectral index, concentration and temperature of each electron species, density and velocity of electron beam and concentration and temperature of the positive ions. Sheath region structure was also investigated in the presence of electron beam and warm positive ions. It was found that the presence of electron beam causes the density distribution of the charged particles in the sheath region as well as the sheath thickness to increase but the potential of the sheath to decrease. Also, it was shown that an increase in the temperature of positive ions leads to a decrease in the density distribution of ions and both cool and hot electron species while the potential of the sheath region increases. Moreover, it was found that an increase in spectral index of the electron species leads to a decrease (an increases) in density distribution of the charged particles (electric potential of the sheath region).
